# Drosophila at the intersection of infection, inflammation, and cancer

**DOI:** 10.3389/fcimb.2013.00103

**Published:** 2013-12-19

**Authors:** Erdem Bangi

**Affiliations:** Department of Developmental and Regenerative Biology, Icahn School of Medicine at Mount SinaiNew York, NY, USA

**Keywords:** Drosophila, innate immune response, inflammation, infection, cancer

## Abstract

Recent studies show that both cellular and humoral aspects of innate immunity play important roles during tumor progression. These interactions have traditionally been explored in vertebrate model systems. In recent years, Drosophila has emerged as a genetically tractable model system for studying key aspects of tumorigenesis including proliferation, invasion, and metastasis. The absence of adaptive immunity in Drosophila provides a unique opportunity to study the interactions between innate immune system and cancer in different genetic contexts. In this review, I discuss recent advances made by using Drosophila models of cancer to study the role of innate immune pathways Toll/Imd, JNK, and JAK-STAT, microbial infection and inflammation during tumor progression.

The interaction between the tumor and the immune system is a complex, multi-step process in which both innate and adaptive branches of the immune system participate (Finn, 2012). The outcome of this antitumor response is variable and unpredictable; it can be tumor suppressive or tumor promoting depending on the immunogenicity and genetic composition of the tumor and the strength of patient's immune response (Finn, 2012). Several recent studies report that at least some aspects of the relationship between the immune system and cancer are also conserved in flies (Pastor-Pareja et al., 2008; Apidianakis et al., 2009; Cordero et al., 2010; Bangi et al., 2012): Drosophila immune system also recognizes and responds to tumors and this response can be tumor promoting or tumor suppressive depending on the genetic composition of the tumor. Here, I briefly summarize studies that use Drosophila to explore the role of the innate immune system during tumor progression.

## Inflammation, tumor associated hemocytes (TAHs), and invasion

The first potential link between cancer and inflammation was proposed 2000 years ago, when the Roman physician Galenos suggested that cancers evolved from inflammatory lesions (Trinchieri, 2012). The first experimental evidence supporting this remarkable observation would not emerge until 1863, when German scientist and physician Rudolf Virchow observed that leukocytes were associated with neoplastic tissues, re-establishing this forgotten link between cancer and inflammation (Balkwill and Mantovani, 2001). Now, it is a well-established fact that inflammation impacts every aspect of of tumor development and progression (Trinchieri, 2012).

An early step in the anti-tumor response is the recruitment of macrophages and other blood cells mediating the innate immune response to the tumor site (Finn, 2012). These cells phagocytose tumor cells and secrete inflammatory cytokines to both maintain the innate immune response and promote and support activation of the adaptive immune response (Finn, 2012). While the Drosophila immune system shows evidence of some primed responses (Kvell et al., 2007), flies lack adaptive immunity as we know it in mammals. However, both the cellular and humoral aspects of the innate immune response and the pathways that mediate them are highly conserved (Hoffmann et al., 1999).

The cellular arm of the Drosophila immune response consists of circulating blood cells called hemocytes. There are three morphologically distinct types of hemocytes in Drosophila that share a common developmental and evolutionary origin with mammalian blood cells (Hartenstein, 2006). Plasmatocytes are the most common hemocyte type in Drosophila, comprising more than 95% of all hemocytes. Plasmatocytes resemble mammalian phagocytes and like macrophages, they are recruited to sites of infections or wounds to phagocytose apoptotic cells, invading microbes, and other foreign bodies (Tepass et al., 1994; Franc et al., 1999; Elrod-Erickson et al., 2000). Like their mammalian counterparts, Drosophila hemocytes are also recruited to epithelial tumors (Pastor-Pareja et al., 2008). Epithelial tumors are often established in Drosophila by generating patches of epithelial cells (clones) mutant for apical/basal polarity genes such as *scrib* (*scr*), *lethal giant larvae* (*lgl*), or *discs large* (*dlg*) while also expressing the oncogenic form of Drosophila *dRas1* (e.g., *scrib*^−/−^ *dRas1*^*V*12^) (Pastor-Pareja et al., 2008; Gonzalez, 2013). Cells mutant for apical/basal polarity genes alone are quickly eliminated from the epithelium by apoptosis in a JNK dependent manner (Rudrapatna et al., 2012). However, co-expressing *dRas1*^*V*12^ in these polarity-defective cells leads to invasive tumors as JNK pathway activation in these tumors promotes MMP expression, basement membrane degradation and invasion instead of apoptosis (Brumby and Richardson, 2003; Pagliarini and Xu, 2003).

Using a *scrib*^−/−^ *dRas1*^*V*12^ tumor model, Pastor-Pareja and collegues showed that hemocytes infiltrate epithelial tumors in Drosophila (Pastor-Pareja et al., 2008). Tumor bearing animals also show increased numbers of circulating hemocytes and enlarged lymph glands as a result of increased hemocyte proliferation. Interestingly, this anti-tumor response is remarkably similar to the immune response to experimentally induced aseptic wounds, consistent with the idea that tumors are like wounds that never heal (Dvorak, 1986).

The mechanism by which hemocytes are recruited to tumors is not clear. However, Tumor Associated Hemocytes (TAHs) are preferentially found in the regions of the tumor where the basement membrane is disrupted (Pastor-Pareja et al., 2008). Basement membrane disruption in the absence of tumors by overexpression of MMP2 is sufficient to induce hemocyte recruitment but not proliferation, indicating that basement membrane break-down is only one of the signals mediating this immune response.

Local activation of JNK signaling in the tumor cells is critical for the maintenance of the anti-tumor response (Pastor-Pareja et al., 2008). JNK signaling promotes the secretion of JAK-STAT activating cytokines (Upd ligands) from the tumor; this initiates a positive feedback loop that activates *upd* expression in hemocytes and the fat body (also the site of antimicrobial peptide expression and release in response to infection). The increased JAK-STAT pathway activity in the hemocytes is required to induce hemocyte proliferation in response to tumors.

Tumor Necrosis Factor (TNF) signaling is another critical component of the inflammatory response activated in response to microbial infection, tissue damage and malignant cells (Waters et al., 2013a). While both tumor suppressive and tumor promoting roles for this pathway have been well established, the molecular mechanisms mediating these different responses are less clear (Waters et al., 2013b). Drosophila has a highly conserved but simplified TNF pathway with a single TNF ligand called Eiger (Egr) (Igaki et al., 2002; Moreno et al., 2002). Removal of *scrib^−/−^* or *lgl^−/−^* cells from the epithelium also requires TNF/Eiger indicating a conserved role for TNF signaling as a tumor suppressor pathway in Drosophila within these genetic contexts (Igaki et al., 2009; Cordero et al., 2010).

**Figure 1:**
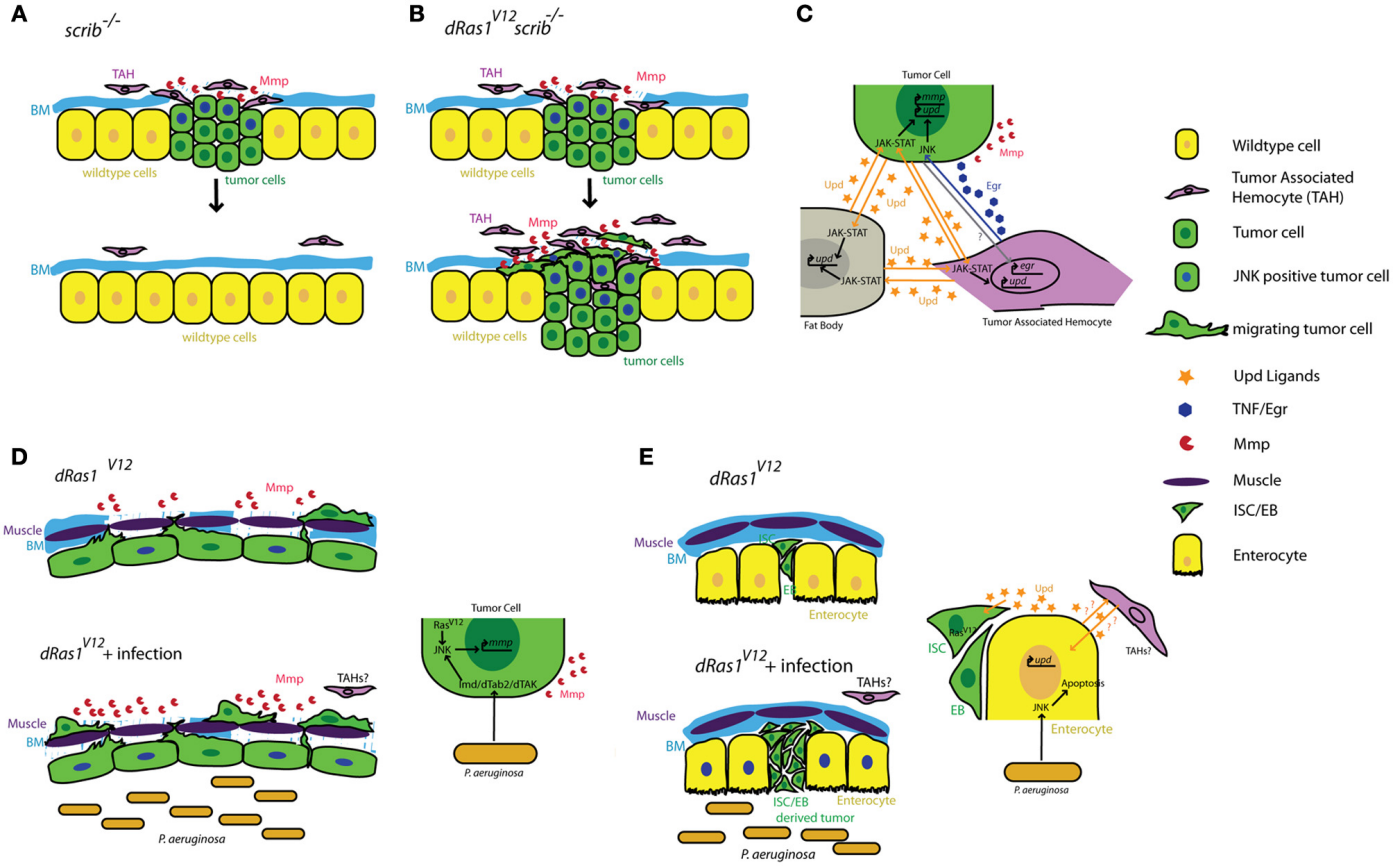
**Summary of tumor cell-immune system interactions in Drosophila.** (**A**) Clones of cells defective for cell polarity genes such as *scrib* (green) generated in the context of a wild type tissue (yellow) are rapidly eliminated from the epithelium with the help of tumor associated hemocytes (TAHs, purple). **(B)** This antitumor immune response is subverted in *scrib*^−/−^ *dRas1*^*V*12^ cells, leading to the establishment of an invasive tumor. **(C)** Reciprocal interactions between tumor cells (green) TAHs (purple), and fat body (gray) result in both local and systemic release of inflammatory cytokines in a positive feedback loop. **(D)** Microbial infection synergizes with *dRas1*^*V*12^ to induce activation of JNK signaling (blue nuclei) and Mmp expression (red) in transformed hindgut epithelial cells, leading to migration and dissemination. **(E)** Microbial infection induces apoptosis of differentiated enterocytes (yellow), and synergizes with with *dRas1*^*V*12^ to induce hyperplastic ISC/EB-like tumors. BM, Basement Membrane; Mmp, Matrix Metalloprotease; ISC, Intestinal Stem Cell; EB, Enteroblast.

Cordero and collegues showed that hemocyte attachment and infiltration of tumors provoke tumor cells to induce high levels of *egr* expression in TAHs (Cordero et al., 2010). By transfusing hemocytes into tumor bearing larvae with *egr* mutant or wildtype hemocytes, Cordero and collegues show that *egr* expression in TAHs is required to induce JNK signaling and MMP expression in tumor cells and that these defects can be partially rescued by transfusing animals with *egr*^+/+^ hemocytes. Most importantly, removing *egr* from TAH's has drastically different consequences on tumors with different genotypes: *scrib*^−/−^ tumors cannot be eliminated from the tissue without *egr*^+/+^ hemocytes, indicating a tumor suppressive role for TAHs and TNF signaling in this genetic context (Figures 1A,C). In contrast, Egr signaling from the TAHs is essential for *scrib*^−/−^ *dRas1*^*V*12^ cells to become invasive tumors (Figures 1B,C) indicating a tumor promoting role for this pathway in this genetic context.

Activation of JNK signaling and induction of MMP1 expression are a part of the normal immune response to facilitate delamination of abnormal cells from the epithelium and promote further infiltration of the wound or infection by hemocytes. As both JNK and TNF pathways are strong inducers of cell death, these MMP expressing cells are normally quickly eliminated by apoptosis to ensure tissue integrity. However, these studies suggest that if these JNK/MMP1 positive tumor cells persist long enough in the tissue, for instance as a result of additional mutations that prevent apoptosis, they can further promote degradation of the basement membrane and infiltration by additional TAHs. This in turn leads to a positive feedback loop that increases the number of JNK/MMP positive cells within the tumor and thereby its metastatic potential (Figures 1A–C).

Aspects of bacterial infection can also be studied by directly expressing pathogen-derived proteins in host tissues. For instance, Drosophila models of *H. pylori* infection have been generated by expressing the *H. pylori* virulence factor CagA in Drosophila tissues (Botham et al., 2008; Wandler and Guillemin, 2012). Certain virulent strains of *H. pylori* possess a secretion system that allows them to directly inject the CagA protein into gastric epithelial cells and can promote the development of gastric carcinoma in a small percentage of infected individuals (Peek and Blaser, 2002; Hatakeyama, 2008; Wroblewski et al., 2010). Wandler and Guillemin showed that CagA expression in discrete domains in the Drosophila wing disc epithelium leads to the activation of apoptosis in a subset of CagA expressing cells in a JNK signaling dependent fashion (Wandler and Guillemin, 2012). Interestingly, loss of *egr* function in the whole animal increased the number of apoptotic CagA expressing cells, but not when *egr* was only reduced in CagA expressing cells. This suggests a non-cell-autonomous role for Egr in apoptotic cell clearance. The authors propose a model whereby loss of Egr from the neighboring wildtype epithelial cells mediate elimination of apoptotic CagA expressing cells from the epithelium. CagA expression also synergized with oncogenic Ras to facilitate JNK mediated tumor progression and invasion, however, the role of Egr in this context has not been investigated. Furthermore, potential roles for the core immune signaling pathways and the cellular immune response in this process remain unexplored possibilities. It will be interesting to see if hemocytes also associate with tumors in this paradigm and whether similar pro-tumor and anti-tumor roles for Egr/TNF signaling can be elucidated.

## Toll/Imd signaling, microbial infection, and cancer

Recognition or pathogen and damage associated molecular patterns by the immune system is a key component of mounting an effective host defense. In Drosophila, this innate immune response is mediated by two pathways: Recognition of Gram positive bacteria and fungi depends on secreted factors that regulate the processing and activation of the Toll receptor ligand Spatzle (Spz) (Lemaitre et al., 1996). Subsequent activation of the Toll pathway leads to the expression and secretion of antimicrobial peptides (AMPs) mediated by NFκB related molecules Dorsal and Dif (Valanne et al., 2011). On the other hand, Gram negative bacteria are recognized by pattern recognition receptors called PGRPs, ultimately leading to activation of another NFκB related molecule called Relish as well as JNK pathway in an Imd dependent fashion (Choe et al., 2002; Ramet et al., 2002; Kallio et al., 2005). In mammals, Toll Related Receptor (TLR) signaling is activated by direct binding of pathogen associated molecules, leading to NFκB-mediated induction of AMP expression (Takeuchi and Akira, 2010). In addition, pathogen associated peptidoglycan fragments are recognized by NOD-like Receptors (NLRs), which leads to activation of NFκB and JNK pathways (Lavelle et al., 2010). Even though there are some differences in the activation mechanisms of these pathways, most of the downstream pathway components and their roles are highly conserved between mammals and Drosophila (see reference 30 for an in-depth comparative analysis).

Stimulation of innate immune responses by microbial components can also modulate migratory potential of epithelial cells (Wang et al., 2003; Merrell et al., 2006) and recent identification of functionally active TLRs in several tumor cell lines point to important roles for TLR signaling in epithelial tumor progression and metastasis (Huang et al., 2005, 2008; Kelly et al., 2006; Rakoff-Nahoum and Medzhitov, 2009). In recent years several groups took advantage of the high degree of conservation of core immune signaling pathways in Drosophila to explore the relationship between innate immune responses and tumor progression.

The gastrointestinal tract is a prominent component of both mammalian and Drosophila immune systems. The intestinal epithelium expresses several TLRs and studies both in murine models and in Drosophila reveal that intestinal epithelial cells respond to microbial infection by secreting AMPs, a Toll/Imd/TLR signaling mediated process (O'Neil et al., 1999; Apidianakis et al., 2005). Interestingly, chronic activation of the immune response is thought to facilitate intestinal tumorigenesis in genetically predisposed individuals (Pasparakis, 2008; Secher et al., 2010), again suggesting a pro-tumorigenic role for Toll/Imd/TLR signaling in the intestine. We found that acute activation of the Imd pathway in response to microbial infection interacts with pre-existing oncogenic mutations to promote tumorigenesis in a *dRas1*^*V*12^ induced model of colon cancer in Drosophila (Bangi et al., 2012). When targeted to the hindgut epithelium—the functional equivalent of the mammalian colon—*dRas1*^*V*12^ activates JNK signaling and MMP expression in a subset of the hindgut epithelial cells. These transformed cells eventually migrate out of the epithelium to colonize distant sites within the animal. While JNK/MMP positive cells do not migrate themselves, both JNK signaling and MMP expression is necessary for the dissemination phenotype. Microbial infection of these animals using a previously established infection paradigm by oral feeding of the Gram negative bacterium *Pseudomonas aeruginosa* (Apidianakis and Rahme, 2009, 2011) leads to a significant enhancement of *dRas1*^*V*12^ induced dissemination in an Imd dependent fashion. Microbial infection in this case increases the metastatic potential of the tumor by increasing the number of JNK/MMP1 positive cells, thereby further compromising the integrity of the tissue and facilitating the migration of *dRas1*^*V*12^ transformed cells (Figure 1D).

By contrast in the midgut, microbial infection synergizes with *dRas1*^*V*12^ to induce intestinal hyperplasia but not invasion or dissemination; in this model, *dRas1*^*V*12^ was targeted to intestinal stem cells (ISCs) and undifferentiated enteroblasts (EBs), the immediate progeny of ISCs (Apidianakis and Rahme, 2009; Pitsouli et al., 2009) (Figure 1E). Hyperplasia is driven by bacteria-induced death of differentiated midgut cells. Curiously, JNK induced secretion of JAK-STAT inducing cytokines (Upd-1, -2, -3) by the dying midgut cells is known to be a key mediator of tissue regeneration (Jiang et al., 2009), reminiscent of the positive feedback loop created between TAHs and tumor cells in the imaginal disc tumor models discussed above (Pastor-Pareja et al., 2008). Adult hemocytes have been reported to respond to microbial infection by phagocytosing invading pathogens in multiple infection paradigms (Elrod-Erickson et al., 2000; Kocks et al., 2005; Nehme et al., 2007). However, there is no evidence that they infiltrate the adult gut as part of the immune response and whether they contribute to hyperplasia and dissemination phenotypes in these intestinal cancer models have not been investigated.

## Antiviral immunity and cancer

In addition to bacterial and fungal infection paradigms, several Drosophila models of viral infection also exist; these include models that use natural viruses that infect Drosophila as well as several viruses that cause disease in humans and those that directly express various viral proteins in Drosophila tissues (Bier and Guichard, 2012; Merkling and van Rij, 2013). The major immune defense against viral infection in insects is the RNA interference pathway, however, several recent reports indicate possible roles for the evolutionarily conserved core immune signaling pathways Toll, Imd, and JAK-STAT in antiviral immunity (Dostert et al., 2005; Zambon et al., 2005; Costa et al., 2009). It would be interesting to combine these viral infection models with available Drosophila cancer models to explore interactions between viral infection, antiviral immunity and cancer.

## Drosophila offers new tools to explore links between immunology and cancer

The presence of an antitumor immune response in Drosophila opens up new avenues of research in the field of tumor immunology. The absence of an adaptive immune response precludes modeling certain aspects of immune response. However, signaling pathways that mediate the interactions between tumor cells and the innate immune system (JNK, JAK-STAT, TNF, Toll/Imd/TLR) as well as the way these pathways interact with each other are highly conserved in flies.

The sophisticated genetic tools available in Drosophila can be used for genetic dissection of conserved aspects of the anti-tumor immune response. For instance, multiple independent targeted and inducible expression systems are available in Drosophila (del Valle Rodriguez et al., 2012), making it possible to separately label and genetically manipulate tumor cells and cells of the immune system. An increasing number of genetically complex tumor models are being reported in Drosophila (Gonzalez, 2013). For instance, 30 multigenic models of colon cancer in the adult Drosophila gut have recently been generated and characterized in our laboratory (Bangi et. al., in review). These models allow us to explore the mechanisms by which the innate immune system reacts to tumors with different genetic compositions.

Lastly, Drosophila is emerging as a useful platform for cancer drug discovery: flies provide a high degree of conservation of cancer relevant pathways as well as appropriate sensitivity to compounds targeting these pathways (Bangi et al., 2011; Gonzalez, 2013). Compound screens in Drosophila using organismal lethality or other complex phenotypic read outs of cancer are revealing new anti-cancer agents with promising activity in mammalian models (Dar et al., 2012). With these tools, Drosophila can be useful both as a genetic model system for tumor immunology but also as a drug discovery platform to screen for compounds that target the immune system and its interactions with tumor cells.

### Conflict of interest statement

The author declares that the research was conducted in the absence of any commercial or financial relationships that could be construed as a potential conflict of interest.
